# The Assessment of Radon Emissions as Results of the Soil Technogenic Disturbance

**DOI:** 10.3390/ijerph17249268

**Published:** 2020-12-11

**Authors:** Timofey Leshukov, Aleksey Larionov, Konstantin Legoshchin, Yuriy Lesin, Svetlana Yakovleva

**Affiliations:** 1 Department of Geology and Geography, Institute of Biology, Ecology and Natural Resources, Kemerovo State University, 6 Krasnaya Street, 650000 Kemerovo, Russia; geo@kemsu.ru; 2 Department of Physiology and Genetics, Institute of Biology, Ecology and Natural Resources, Kemerovo State University, 6 Krasnaya Street, 650000 Kemerovo, Russia; larionov@kemsu.ru; 3 Department of Mine Surveying and Geology, Mining Institute, T.F. Gorbachev Kuzbass State Technical University, 28 Vesennaya street, 650000 Kemerovo, Russia; lyuv.geo@kuzstu.ru; 4 Department of Ecology and Nature Management, Institute of Biology, Ecology and Natural Resources, Kemerovo State University, 6 Krasnaya Street, 650000 Kemerovo, Russia; ecology@kemsu.ru

**Keywords:** radon flux density, indoor radon, underground coal mining, radon genotoxicity, clastogenic/aneugenic effects

## Abstract

^222^Rn is a specific indoor-type pollutant that represents a primary radiological hazard as a main source of ionizing radiation (IR) for humans. Coal mining creates new sources of gas that are formed over mines. This process can significantly increase the density of radon flux. Therefore, the concentration of radon in a room can increase. We investigated the territory of the Leninsk-Kuznetsky district of the Kemerovo region, which is subject to underground mining. Two groups of residential locations and measuring points of radon flux density were selected to identify the higher emanation relationship of radon and mining-affected areas. The first group (Case group) included subjects located within the territory of the underground mine; the other (Control group) included subjects in an area without mining. Radon flux density in coal mining areas was significantly higher than in the rest of the territory; moreover, the percentage of values in the Case group that had a radon flux density above 80 mBq·m^−2^·s^−1^ was 64.53%. For the Case group, 20.62% of residential buildings had a radon concentration above 200 Bq/m^3^. For the studied area, the radon flux density correlates positively (r = 0.79, *p* = 0.002) with indoor radon. Additional clastogenic/aneugenic effects are also found in dwellings with increased volume activity of radon (VAR) within the territories of underground mines. Ring chromosomes are positively correlated with radon levels in smoker groups but not in non-smokers. An increased frequency of binucleated (BN) cells with micronuclei (MN) is also positively correlated with VAR regardless of smoking status. It has been concluded that reducing the total exposure level of a population to radon can be achieved by monitoring areas with underground mines where radon is emitted heavily.

## 1. Introduction

Radon is a product of the radioactive decay of isotope series, starting with natural ^238^U, ^232^Th, and ^235^U dispersed in the earth’s crust. A relatively long-lived isotope of Rn is ^222^Rn (radon), the daughter-element of ^238^U series, and the immediate precursor is radium with T_1/2_ = 3.82 days. It can accumulate in dwellings in significant concentrations and represents a significant radiological hazard as a primary source of ionizing radiation to humans [1]. 

^222^Rn has a relatively short lifetime, and its presence in outdoor air is very minimal. Thus, it is considered an indoor-type pollutant. The problem of resident radon exposure was identified after this discovery. Its carcinogenic effect was known from the work of miners in uranium mines long before [2]. The overall risk assessment of mortality from exposure to radon was 9% of all lung cancer deaths in the European population [3]. An extensive study, including an analysis of articles from 1980 to 2016, was conducted to assess the total burden of additional lung cancer incidences caused by the influence of radon. The average number of years of life lost as a result of residential radon calculated for Canada was 0.066 years for non-smokers and 0.198 years for past/current smokers [4]. Many countries recognize the lack of effort to reduce indoor radon, as well as the need for relevant countermeasures [5].

Therefore, we have strong evidence of the adverse effects of radon in the population according to the disability-adjusted life year (DALY) loss as significant data for investigating the molecular mechanism of health damage. Inhaled radon and its short-lived progeny emit α-particles (^218^Po and ^214^Po) and cause lung-cell irradiation [6]. Some amounts of radon can be dissolved in blood and reach vulnerable targets, such as bone marrow cells [7]. The primary target for ionizing radiation is DNA. In the case of high-linear energy transfer (LET), many lesions are distributed within small sites (clusters) causing specific cluster DNA damage; the higher the LET of IR, the more lesion counts are in such clusters [8].

Chromosomal aberrations (CAs) and micronuclei in cytochalasin-blocked (CBMN) cells reflect an individual’s damage from different genotoxic factors and can be markers of genotoxic risk [9]. Cytogenetic indicators are also useful biomarkers for carcinogenic responses (including the influence of radiation) in occupational and domestic conditions [10,11].

The relationship between soil gas radon concentration and geological structure is well known. Soils contain different volumes of parent elements for radon (U, Th, and Ra), which determines their radioactivity [12,13,14,15]. Tectonic disturbances are expressed in the form of linear anomalies of a high soil gas radon concentration. In addition, they differ in violations of different ages and types [16,17,18]. Dynamically-active fault lines exhibit a higher radon emission. Additionally, tectonic disturbances associated with compression zones are more related to lower VAR values than to the spreading zones. Soil moisture content and porosity also influence the diffusion of radon through soil [19].

In addition to these natural factors, underground coal mining works create new transport routes for gas, which are formed during the collapse of mine roofs. Residential buildings in the regions of long-standing coal mining enterprises are often located in the vicinity of mines, which may increase human exposure to radon. This process has been studied in the coal mining regions of Great Britain, Germany, Poland, Ukraine, etc. [20,21,22,23]. High concentrations of radon in soil air in all studies of similar areas in the UK, Germany, Poland, and Ukraine have been recorded. Ambiguous results have been obtained in residential buildings. In some cases, excesses have been recorded only in the basements of buildings, while in others, excesses have been recorded throughout the living areas as well. This data allow the authors to suggest their connection with the design features of the studied objects. The effect of underground mines on the concentration of radon in residential buildings may have regional aspects (geological, social, medical, and others). This problem arises especially in hemiboreal/boreal climate conditions supported by the development of energy-saving construction technologies aimed at improving thermal insulation properties [24].

The main objective of this study is to present the influence of underground mining on the flux density of radon and indoor radon to evaluate the possible long-term effects as additional areas of genotoxicity for local populations. Moreover, this study examined a territory in which coal mining was completed more than 10 years ago. This fact must be evaluated to rationally use such territories in this region and throughout the world. In previous studies in the UK, Germany, and Poland, the territories of working mines have also been studied.

## 2. Material and Methods

### 2.1. Study Location

In ArcGIS 10.3.1, maps were constructed of the geological structure of the study area, the location of the underground mines, and large tectonic disturbances. The object of research was the Leninsk-Kuznetsky district of the Kemerovo region. The population of the study area is 94,398 people in 2020. The tectonic structure of the research site is represented by the tectonic blocks and divided by tectonic disturbances. The rocks are represented by the Kazancovo–Markinskaya, Uskatskaya, Leninskaya, and Osinovskaya formations composed of a series of interbedded siltstones, sandstones, mudstone, and coal. In river valleys, they are covered by quarter sediments of the Inya River mainly consisting of loam. The main faults of the study area with complex kinematics are Kilchigizsky, Zhurinsky (Sokolovsky), and Vinogradovsky, which are also accompanied by a concomitant zone of tectonic disturbance of varying length along the strike and drop zones. The tectonic disturbance zone of the region determines the presence of transporting channels for radon, and its progeny is formed as a result of the decay of radium and uranium in the rock.

The soils of the study area are characterized by significant homogeneity and are represented by leached chernozem.

**Figure 1:**
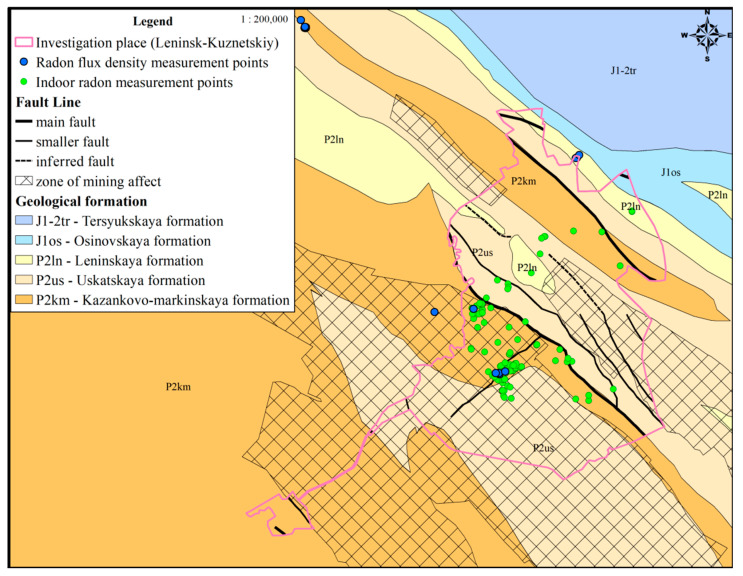
Geological map of the Leninsk-Kuznecky district with zone of mining affect.

The presence of underground mines, which cover a significant territory of the Leninsk-Kuznetsky district (Figure 1), and active mining can form new potential entry routes of radon to the surface. Mining-affected territories consist of tension and compression zones, which differ in radon flux density and lifetime.

### 2.2. Methods

#### 2.2.1. Indoor Radon and Radon Flux Density

These studies were carried out from November 2018 to February 2019. The radon level was studied by a Camera-01 device (STC Niton, Moscow, Russia), using a passive adsorption method with SK-13 charcoal sorption columns. Two absorber-columns were installed in a room at a height of 0.7–1.3 m above the floor for 6 to 7 days to obtain an integral VAR indicator to eliminate the effect of short-term changes in the VAR; such as, for example, ventilation before and during research. Absorber-columns were installed in the rooms where inhabitants spent a longer time (usually, a bedroom or the kitchen). VAR levels were considered to be the average obtained by two absorbers to exclude the influence of circulation air processes in buildings.

The houses in the study area were built in different years from 1924 to 2013, excluding the possibility of the effect of the engineering and construction features on the radon-accumulating properties of buildings. The study involved wooden and brick houses with wooden floors and cellars under each of them. A total of 120 residential buildings were studied. The study of indoor radon concentration was carried out from December 2018 to February 2019. Average monthly climate data: December—average temperature −9.0°, pressure—1024 hPa, relative humidity—88%; January—average temperature −10.2°, pressure—1023 hPa, relative humidity—87%; February—average temperature—−6.7°, pressure—1024 hPa, relative humidity—86%.

The average height above sea level within the study area varies in the range of 200 to 280 m. The measurement of the radon flux density was carried out in September 2019. Average monthly climate data: average temperature +10.7°; pressure—1015 hPa, relative humidity—70%. Samples were taken in the range of air temperatures 14.58 ± 5.16 °C, pressures—1016 ± 4.6 hPa, humidity—59.64 ± 13.06%. The temperature was acceptable for measurements according to the method, with an error not exceeding 30%.

Radon flux densities were measured using charcoal sorption columns (SK-13) and cameras (NK-32). The cameras were pressed firmly into the loose soil. Upon collection two to eight hours later, the cameras were returned to the laboratory and studied within 12 h after being resealed. A total of 687 individual readings of radon flux density were obtained. Of these, 375 were obtained from the territories of underground mines. Radon flux densities were measured in dry day conditions to exclude the influence of water saturation on soil.

In addition, we carried out duplicate measurements of the radon flux density at the measurement points of the radon flux density and indoor radon. According to these results, the data differed from the primary measurements by no more than 5–10%.

We used the limits of radon concentration inside residential buildings—200 Bq m^−3^ and the density of radon flux from the soil—80 mBq m^−2^ s^−1^, which, according to the radiation standards of the Russian Federation, do not require additional radon protection measures. To investigate the influence of underground mines as a predictor of radon flux densities and higher indoor radon, data were classified into two groups. The first group (Case group) included measurements from territories located on underground mines. Other radon flux density measurements were assigned to the second group (Control group).

To investigate the connection between the measured radon flux density near and under buildings, ten measurements of radon flux density were carried out for each dwelling, and the true value was considered the average of these measurements. For correlation analysis, 12 buildings and their territories were used.

#### 2.2.2. Blood Samples Group Description

Samples of peripheral blood were collected from 50 residents of the investigated district, who were inhabitants of low-stage dwellings with different VAR levels (Table 1). All participants signed an “informed agreement” form describing the research aims, and this was performed in accordance with the requirements of the Ethics Committee of the Kemerovo State University. The criteria for including a participant was as follows: living in an investigated home for no less than 2 years; age 18–60 years; no working under harmful conditions such as coal mining, the chemical industry, etc.; no ongoing antibiotic or antiviral therapy or chronic inflammation disease; no X-ray or other radiation exposure; no plane flights within 3 months of blood sampling. After all these restrictions, we used 50 samples that contained 0.05% of the town’s population.

#### 2.2.3. Cytogenetic Methods

CAs and a CBMN assay in peripheral blood lymphocyte (PBL) were used as the biological marker of genotoxicity. Both methods used a RPMI-1640 (PanEco, Moskow, Russia) growth medium, a fetal bovine serum (HyClone) at a final concentration of 15%, and 100 U/mL penicillin–streptomycin (PanEco, Moskow, Russia). Phytohemagglutinin (PHA) was used at a final concentration of 1.5% for CAs and 2% for CBMN. The growth conditions were 37 °C and 5% CO_2_. PHA stimulated the mitotic division of lymphocytes with a following interruption of the cell cycle during the second division. For the CAs assay, cultures were exposed with colchicine during 48–50 h of cultivation, and for the CBMN assay, cytochalasin stopped mitosis at the cytokinesis stage (exactly 44 h from culture initiation).

For CAs, after a 48 h incubation period, 0.5 μg/mL of colchicine was poured into each culture. Subsequently, each culture was treated with a hypotonic solution (KCl, 0.55%) and fixed using Clarke’s solution (3 volumes of methanol and 1 volume of glacial acetic acid) (Ricoul et al., 2017) [25]. For analysis, BN cells were scored according to the following criteria: two nuclei in a BN were placed within the same cytoplasm boundaries and nuclear membranes were intact and distinguishable from either nucleus. Both nuclei have equal size and staining intensity, and are unconnected from each other or attached with a nucleoplasmatic bridge no wider than 1/4th of the nuclei diameter. A total of 1000 BN cells were analyzed in each slide, and 500 cells were scored to evaluate the frequency of cells with 1, 2, 3, and 4 nuclei. The nuclear division index (NDI) was calculated as NDI = [M1 + 2(M2) + (M3) + 4(M4)]/N, where M1–M4 = numbers of cells with one to four nuclei and N is the total number of scored cells. 

Quantification of the aberrations was performed using light microscopy at 1000× magnification (oil immersion) without karyotyping. The selection of metaphases included in the analysis and criteria for cytogenetic abnormalities conformed to the generally accepted recommendations [26].

For the CBMN after a 44 h incubation period, 6 μg/mL of cytochalasin B (AppliChem, Council Bluffs, USA) was added to each flask and incubated for another 24 h. After this, each culture was treated with a 0.075 KCl solution and fixed with Clarke’s solution [27].

#### 2.2.4. Statistical Analysis

Statistical analysis was performed using the program StatSoft STATISTICA 12.0. We used the Kolmogorov–Smirnov test to verify the compliance of the data with the normal distribution. Data analysis was performed using the non-parametric statistics block. Group comparisons were performed using the *U*-rank Mann–Whitney test. The Spearman correlation coefficient for non-parametric data was used to calculate the correlation between clustogenic and aneugenic effects and residential VAR levels, as well as between the radon flux density and indoor radon.

## 3. Results and Discussion

### 3.1. Relationship between Underground Mines and Radon Flux Densities

The main radon flux density data collected from the Leninsk-Kuznetsky district are presented in Table 2. The average radon flux densities in the Case group were four and nine times higher than the indicator for the Control group and studied geological formations, respectively. The *U*-rank Mann–Whitney test showed significant differences between the Control and Case groups (*p* < 0.0001). In addition, other data are also substantially higher in the Case group. The radon flux density in the Case group varied from 8 to 3310 mBq·m^−2^·s^−1^, much wider than in the Control group. The percentage of values in the Case group that had a radon flux density above 80 mBq·m^−2^·s^−1^ was 64.53%; therefore, radon-protective measures are needed for most erected buildings in the territory. Despite the fact that low values were also present in the territory, they were determined mainly in places of clay soils found in demolished houses. High concentrations of methane and carbon dioxide led to their demolition. On the one hand, radon flux densities may be associated with the spontaneous combustion of a coal seam [28,29], on the other hand, they may be associated with dynamic processes above mines [22,30]. Thus, the territory atop underground mines is characterized as hazardous for residential use owing to radiation risk.

### 3.2. Relationship between Underground Mines, Indoor Radon, and Radon Flux Density

The base statistics for the indoor radon data in Leninsk-Kuznetsky are presented in Table 3. The mean indoor radon for the Case group was higher than that of the Control group. However, the differences were not as significant as those in the radon flux density. Thus, this may be related to the influence of the construction and material features of buildings. For example, R. Klingel and J. Kemski (1999) obtained significant differences only for basements. Alternatively, T.K. Ball and M. Wysochka (2011; 2016) revealed significant differences for the first floors of buildings.

For the Case group, 20.62% of the residential buildings with a radon concentration above 200 Bq/m^3^ were detected, significantly exceeding the typical levels for Russia as well as other countries. In addition, indoor radon in the Control group also exceeded these values, although not significantly. This result can be explained by the direct and indirect effects of mining. A direct influence is associated with the cracks in rocks that form as a result of underground mining. As a result, the radon flux density increases. The indirect influence is associated with the location of underground mining, which is usually produced in areas of less disturbed rocks. Thus, a large number of houses in the Control group are located in zones of tectonic disturbance of rocks. These tectonic faults are less active than disturbances formed by underground mining. Therefore, this affects their radon output. In addition, the crack zones of the mining area are incomparably more common than in areas of natural disturbances. However, improving the insulating properties of residential buildings will lead to a significant increase in indoor radon [24].

For the studied territory, radon flux density correlates positively (r = 0.79, *p* = 0.002) with indoor radon, confirming mainly impact geogenic radon. For both groups, the radon flux density determines the concentration of radon in a residential building.

### 3.3. Clustogenic and Aneugenic Effects and Residential VAR Level

The frequency of chromosomal aberration did not significantly differ between males and females and slightly increases in the smoker group. At the same time, the frequency of 2-nucl. cells with MN (in most cases this parameter contains one micronucleus binucleated cell) increased in the female group and also in the “Nonsmokers” group. But, “Nonsmokers” mostly contained females (Table 4). It also should be noted that there is a high rate of “acentric fragments” among the residents of the city of Leninsk-Kuznetsky (0.96%) in comparison to the mean value of the Kemerovo region (0.58%) obtained in early research. This possibly reflects an increase in specific genotoxic factors in Leninsk-Kuznetsky. We also compared CAs and MN levels with the relevant group of people who lived in Kemerovo without underground mines in the surrounding area and found no correlation between radon levels and any biomarkers.

**Figure 2:**
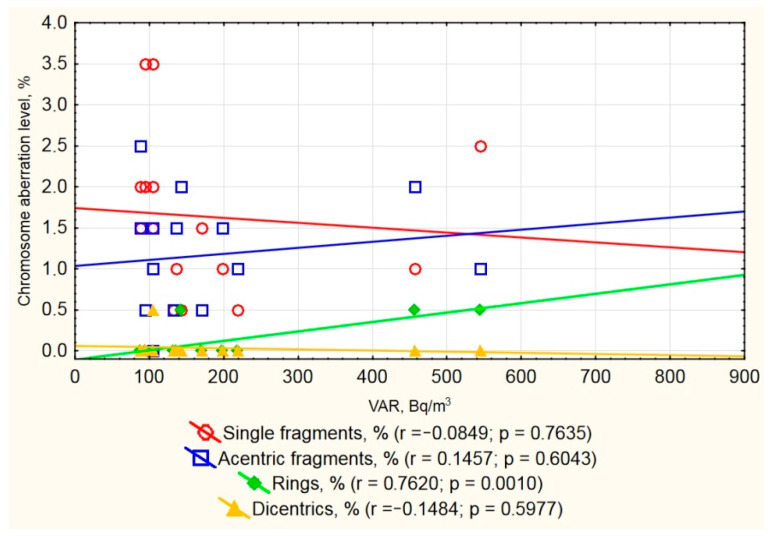
Frequency of chromosome aberrations and residential volume activity of radon (VAR) in studied inhabitants.

We also investigated a correlation between residential VAR and cytogenetic abnormality frequency in the observed group. We have not found any such correlation in the non-smoker group. However, amongst smokers, we found that increased radon levels were accompanied by an increase in ring chromosome frequency (Figure 2). This type of chromosomal aberration is a biological marker of the effect of radiation on the body, including high LET radiation. Many studies prove the relationship between radon and cigarette smoke, as well as their synergistic effect, which increases the risk of lung cancer in smokers. For example, a combined study showed the multiplicative effect of radon along with smoking and the development of lung cancer, confirming the hypothesis of a synergistic effect of the two factors [31]. In vitro experiments have shown a higher rate of dicentrics in smokers’ cells exposed to radon [32].

**Figure 3:**
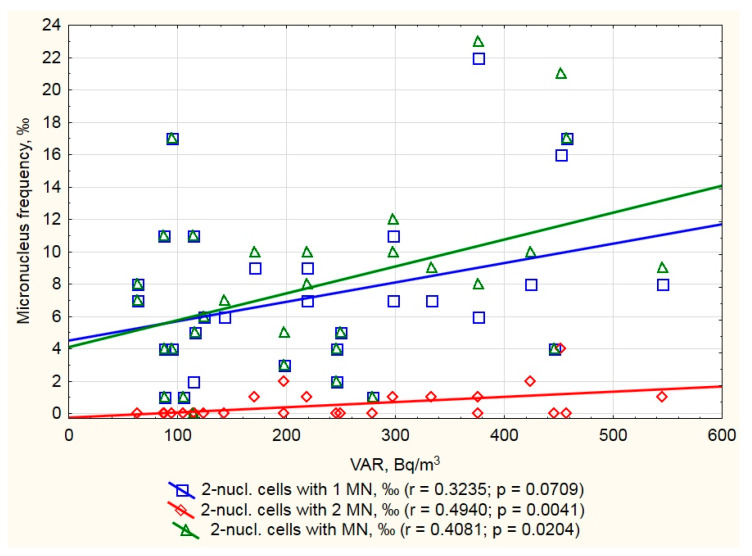
Frequency of peripheral lymphocytes with micronuclei (MN) and residential VAR in studied inhabitants.

Considering the CBMN assay, we found that the frequency of BN cells with any amount of MN had a positive correlation with VAR in a dwelling; “3 MN” and “>3 MN” BN cells were not included in this scatterplot because of their isolated encounters among analyzed cells (Figure 3). Thus, we suggest that CBMN in PBL provides a good biomarker even in low-dose residential radon exposure. Additionally, some research has found that CBMN correlates with radon exposure [33,34] although, at the same time, some similar research has found no such correlation [35].

## 4. Conclusions

This work obtained evidence of significant differences in the density of radon flux between soils located within mines and outside their boundaries. The observed relationship between high radon flux density and underground mines is important in identifying areas where radon preventive measures need to be taken in future construction and in completed structures.

At the same time, according to our study, the location of a residential building within the area of an underground mine does not always determine the high level of radon, but mining is the main risk factor. The identification of houses with elevated radon levels can be facilitated by creating monitoring zones in these areas.

This work did not obtain statistically significant differences in the radon concentration inside the premises of the Case group and the Control group, which, based on the huge difference in the radon flux density, makes us pay attention to other factors of radon accumulation.

Studies of genotoxic effects in residents of the surveyed territories indicate a possible effect of the formation of cytogenetic abnormalities (for example, an increase in ring chromosomes in a group of smokers) and the possibility of using these biomarkers in similar environmental studies.

## Figures and Tables

**Table 1:** Gender and age characteristics of observed persons.

Parameters	Total (Smokers-Nonsmokers)	Male (Smokers-Nonsmokers)	Female (Smokers-Nonsmokers)
Number	50 (23–27)	20 (15–5)	30 (8–22)
Age (m ± SE)	42.4 ± 2.58	36.2 ± 4.58	45.0 ± 3.51
Age (min–max)	24–58	24–56	28–58

Note: m—mean; SE—standard error.

**Table 2:** The radon flux density for investigated areas.

Radon Flux Density, mBq·m^−2^·s^−1^								
Samples	N	Average	Median	Range	Percentage > 80	Percentage > 200	10pc	90pc
P_2_km	178	22.87 ± 1.01	20	10–128	0.56%	0%	20	45
P_2_ln	134	46.61 ± 3.62	27	9–260	19.4%	1.49%	48	110
Case group	375	181.59 ± 13.32	119	8–3310	64.53%	26.4%	37	770
Control group	312	33.07 ± 1.78	22	9–260	8.65%	0.64%	17	111

Note: P_2_km—Kazankovo–Markinskaya formation; P_2_ln—Leninskaya formation.

**Table 3:** The indoor radon for investigated areas.

Samples	N	Average, Bq/m^3^	Median, Bq/m^3^	Range, Bq/m^3^	Percentage ≥200, Bq/m^3^	Percentage ≥400, Bq/m^3^	10pc, Bq/m^3^	90pc, Bq/m^3^
All homes	120	159.45 ± 17	121.38	15.75–1715	18.34%	4.17%		
Case group	97	168.57 ± 20.66	127.25	15.75–1715	20.62%	5.15%	47.25	272.5
Control group	23	120.97 ± 14.79	101	24.75–304.25	8.70%	0	84.75	119

**Table 4:** Chromosomal aberrations (CAs) and micronuclei in cytochalasin-blocked (CBMN) frequency in the examined group.

Parameters	Leninsk-Kuznetsky (N = 50)	Male (N = 20)	Female (N = 30)	Smokers (N = 21)	Nonsmokers (N = 29)
Numbers of metaphases	10,000	4000	6000	4200	5800
Total CAs, % (95% CI)	2.98 (2.56–3.39)	2.83 (2.08–3.59)	2.84 (2.26–3.42)	3.05 (2.39–3.72)	2.68 (2.04–3.32)
Single fragments, % (95% CI)	1.81 (1.48–2.13)	1.79 (1.00–2.59)	1.65 (1.22–2.07)	1.69 (1.14–2.24)	1.68 (1.17–2.19)
Chromatide exchanges, % (95% CI)	0.03 (0–0.07)	0.04 (0–0.13)	0.03 (0–0.07)	0.06 (0–0.14)	0.02 (0–0.06)
Acentric fragments, % (95% CI)	0.96 (0.76–1.15)	0.88 (0.57–1.18)	1.02 (0.75–1.28)	1.11 (0.80–1.43)	0.88 (0.59–1.17)
Dicentric, % (95% CI)	0.10 (0.02–0.18)	0.17 (0–0.37)	0.04 (0–0.12)	0.14 (0–0.28)	0.04 (0–0.12)
Rings, % (95% CI)	0.08 (0.03–0.14)	0	0.10 (0.02–0.17)	0.08 (0–0.19)	0.06 (0–0.13)
NDI	2.00 (1.85–2.15)	2.08 (1.90–2.26)	1.95 (1.71–2.19)	2.12 (1.94–2.30)	1.91 (1.67–2.15)
2-nucl. cells with MN, ‰ (95% CI)	7.34 (5.65–9.04)	5.33 (2.42–8.24)	8.32 * (6.30–10.35)	5.45 (3.11–7.80)	9.08 * (6.83–11.33)
2-nucl. cells with 1 MN, ‰ (95% CI)	6.81 (5.35–8.26)	5.13 (2.57–7.69)	7.67 * (5.90–9.45)	5.31 (3.24–7.39)	8.25 * (6.26–10.24)
2-nucl. cells with 2 MN, ‰ (95% CI)	0.49 (0.23–0.75)	0.53 (0.07–1.00)	0.48 (0.16–0.81)	0.45 (0.07–0.84)	0.54 (0.17–0.91)
2-nucl. cells with 3 MN, ‰ (95% CI)	0.13 (0.01–0.25)	0.13 (0–0.34)	0.13 (0–0.28)	0.09 (0–0.26)	0.17 (0–0.33)
2-nucl. cells with >3 MN, ‰ (95% CI)	0.11 (0.02–0.20)	0.13 (0–0.30)	0.10 (0–0.21)	0.09 (0–0.23)	0.13 (0–0.26)

Note: Differences in Mann–Whitney U-test * *p* < 0.01.
